# Implementation and acceptability of a heart attack quality improvement intervention in India: a mixed methods analysis of the ACS QUIK trial

**DOI:** 10.1186/s13012-019-0857-7

**Published:** 2019-02-06

**Authors:** Kavita Singh, Raji Devarajan, Padinhare P. Mohanan, Abigail S. Baldridge, Dimple Kondal, David E. Victorson, Kunal N. Karmali, Lihui Zhao, Donald M. Lloyd-Jones, Dorairaj Prabhakaran, Shifalika Goenka, Mark D. Huffman, on behalf of the ACS QUIK Investigators, G. Vijayaraghavan, Geevar Zachariah, J. Rajan, S. Sivasankaran, Dorairaj Prabhakaran, Donald M. Lloyd-Jones, K. Srinath Reddy, S. Harikrishnan, Robert O. Bonow, Darwin R. Labarthe, Sidney C. Smith, Brahmajee Nallamothu, Thomas Alexander, Karla Hemming, Simon Thom, K. R. Sundaram, Lawton Cooper, Lihui Zhao, Mark D. Huffman, P. P. Mohanan, Raji Devarajan, Abigail S. Baldridge, Dimple Kondal, Mumtaj Ali, Divin Davies, Prasad Arumugan, T. C. Aneesh

**Affiliations:** 1 Centre for Chronic Disease Control, New Delhi, India; 2 Public Health Foundation of India, Gurgaon, India; 3 Westfort Hi-Tech Hospital, Ltd, Thrissur, India; 4 Cardiological Society of India – Kerala Chapter, Kerala, India; 5 Department of Medicine, Northwestern University Feinberg School of Medicine, 680 N. Lake Shore Drive, Suite 1400, Chicago, IL 60611 USA; 6 London School of Hygiene and Tropical Medicine, London, UK; 7 Indian Institute of Public Health-Delhi, New Delhi, India

## Abstract

**Background:**

The ACS QUIK trial showed that a multicomponent quality improvement toolkit intervention resulted in improvements in processes of care for patients with acute myocardial infarction in Kerala but did not improve clinical outcomes in the context of background improvements in care. We describe the development of the ACS QUIK intervention and evaluate its implementation, acceptability, and sustainability.

**Methods:**

We performed a mixed methods process evaluation alongside a cluster randomized, stepped-wedge trial in Kerala, India. The ACS QUIK intervention aimed to reduce the rate of major adverse cardiovascular events at 30 days compared with usual care across 63 hospitals (*n* = 21,374 patients). The ACS QUIK toolkit intervention, consisting of audit and feedback report, admission and discharge checklists, patient education materials, and guidelines for the development of code and rapid response teams, was developed based on formative qualitative research in Kerala and from systematic reviews. After four or more months of the center’s participation in the toolkit intervention phase of the trial, an online survey and physician interviews were administered. Physician interviews focused on evaluating the implementation and acceptability of the toolkit intervention. A framework analysis of transcripts incorporated context and intervening mechanisms.

**Results:**

Among 63 participating hospitals, 22 physicians (35%) completed online surveys. Of these, 17 (77%) respondents reported that their hospital had a cardiovascular quality improvement team, 18 (82%) respondents reported having read an audit report, admission checklist, or discharge checklist, and 19 (86%) respondents reported using patient education materials. Among the 28 interviewees (44%), facilitators of toolkit intervention implementation were physicians’ support and leadership, hospital administrators’ support, ease-of-use of checklists and patient education materials, and availability of training opportunities for staff. Barriers that influenced the implementation or acceptability of the toolkit intervention for physicians included time and staff constraints, Internet access, patient volume, and inadequate understanding of the quality improvement toolkit intervention.

**Conclusions:**

Implementation and acceptability of the ACS QUIK toolkit intervention were enhanced by hospital-level management support, physician and team support, and usefulness of checklists and patient education materials. Wider and longer-term use of the toolkit intervention and its expansion to potentially other cardiovascular conditions or other locations where the quality of care is not as high as in the ACS QUIK trial may be useful for improving acute cardiovascular care in Kerala and beyond.

**Trial registration:**

NCT02256657

**Electronic supplementary material:**

The online version of this article (10.1186/s13012-019-0857-7) contains supplementary material, which is available to authorized users.

## Background

India is estimated to have the largest number of fatal acute myocardial infarctions in the world because of its combination of relatively high incidence and case fatality rates and its large population [1]. In high-income country settings, implementation of guideline-based treatment has been associated with improvements in clinical outcomes. For example, the CRUSADE (Can Rapid Risk Stratification of Unstable Angina Patients Suppress Adverse Outcomes With Early Implementation of the American College of Cardiology (ACC)/American Heart Association (AHA) Guidelines) National Quality Improvement Initiative demonstrated that a 10% higher adherence to clinical performance measures across 350 hospitals studied between 2001 and 2003 (*n* = 64,775 patients) was associated with a 10% lower adjusted odds of in-hospital mortality [2]. In countries like Brazil and China, randomized trials have demonstrated improvements in process measures using quality improvement toolkit interventions that include clinician education, reminders, and case manager training [3–5]. However, these trials were not powered to demonstrate improvements in clinical outcomes.

In the Indian context, data collected from 2007 to 2009 for the Kerala ACS Registry (*n* = 25,748 patients) demonstrated gaps in optimal medical care as well as inappropriate care, such as delayed thrombolysis for patients presenting with non-ST-segment elevation myocardial infarction [6, 7]. Although pilot studies have demonstrated potential benefits of implementing quality improvement strategies [8], large-scale studies have not been carried out in India until recently. To address this gap in knowledge, the Acute Coronary Syndrome Quality Improvement in Kerala (ACS QUIK) cluster randomized, stepped-wedge trial was designed to develop, implement, and evaluate a quality improvement toolkit intervention to improve processes of care and clinical outcomes for patients with acute myocardial infarction [9]. The ACS QUIK trial demonstrated improvements in process of care measures but did not demonstrate a reduction in the 30-day rate of major adverse cardiovascular events, defined by all-cause mortality, reinfarction, stroke, or major bleeding, in the context of improving background care during the trial. There was expected heterogeneity in the primary outcome across the participating hospitals.

We performed a mixed methods process evaluation of the ACS QUIK trial to improve the understanding of the contextual factors that affected implementation, the relative usefulness of the toolkit intervention, and how the interaction of these influenced study outcomes. Mixed methods research employs rigorous quantitative and qualitative research involving multiple types of data (survey questionnaire, in-depth oral interviews, text-messages) to maximize the strengths and counterbalance the weaknesses of each data type and aids in real-life contextual understanding of a research problem from multi-level perspectives [10, 11]. Process evaluations using mixed methods are useful in multicenter trials involving complex interventions to explore physicians’ views of the intervention, understand which components of the intervention worked, and evaluate variation of the intervention effects among sub-groups. Exploring the context and potential mechanisms of action and relating these data with trial outcomes provide further evidence on the potential utility of the toolkit intervention. This has relevance for potential adaptation of the ACS QUIK toolkit intervention in other low-resource settings given the contextual differences and challenges in implementing the guideline-based treatments, which have been shown to be effective in high-income country settings.

This paper presents the development, implementation, acceptability, sustainability, facilitators, barriers, and context of the ACS QUIK toolkit intervention using the United Kingdom’s Medical Research Council framework for evaluating complex interventions [12]. Our aim was to understand key findings from the ACS QUIK trial from the perspectives of physicians:

1. How was the toolkit intervention developed?
2. What facilitators, barriers, and context were important for the toolkit intervention’s implementation and local applicability?
3. What was the acceptability of the toolkit intervention among physicians?
4. What was the interaction between context and underlying mechanisms to support the trial results (i.e., how and why the toolkit intervention improved process of care measures but not clinical outcomes?)?

## Methods

### Overview

This mixed methods study included pre-trial toolkit intervention development (March 2011) using semi-structured interviews and focus group discussions and within-trial online survey data and semi-structured interviews with physicians (November 2015–December 2016) involved in the ACS QUIK trial. The analysis used both survey and interview responses to identify key factors influencing the success of the toolkit intervention components and to understand the interactions (underlying mechanisms) among the toolkit intervention components, local context, and trial outcomes.

### Research setting

The ACS QUIK study methods have been described [9]. In brief, the trial evaluated the effect of a quality improvement toolkit intervention based on American Heart Association’s Get With the Guidelines program across 63 participating hospitals in Kerala, India, using a cluster randomized, stepped-wedge trial design. Participating hospitals included private (*n* = 42, 67%), government (*n* = 9, 14%), and nonprofit (*n* = 12, 19%) hospitals. Each hospital identified at least two members of the quality improvement team to participate in this trial. Between November 2014 and November 2016, 21,374 eligible patients presenting with acute myocardial infarction were enrolled in the study. The primary outcome was the rate of major adverse cardiovascular events, defined as all-cause mortality, recurrent myocardial infarction, stroke, or major bleeding, at 30 days between the intervention and control groups, adjusted for clustering and temporal trends.

### Design and implementation of the ACS QUIK toolkit intervention

#### Formative work for toolkit intervention development

**Fig. 1:**
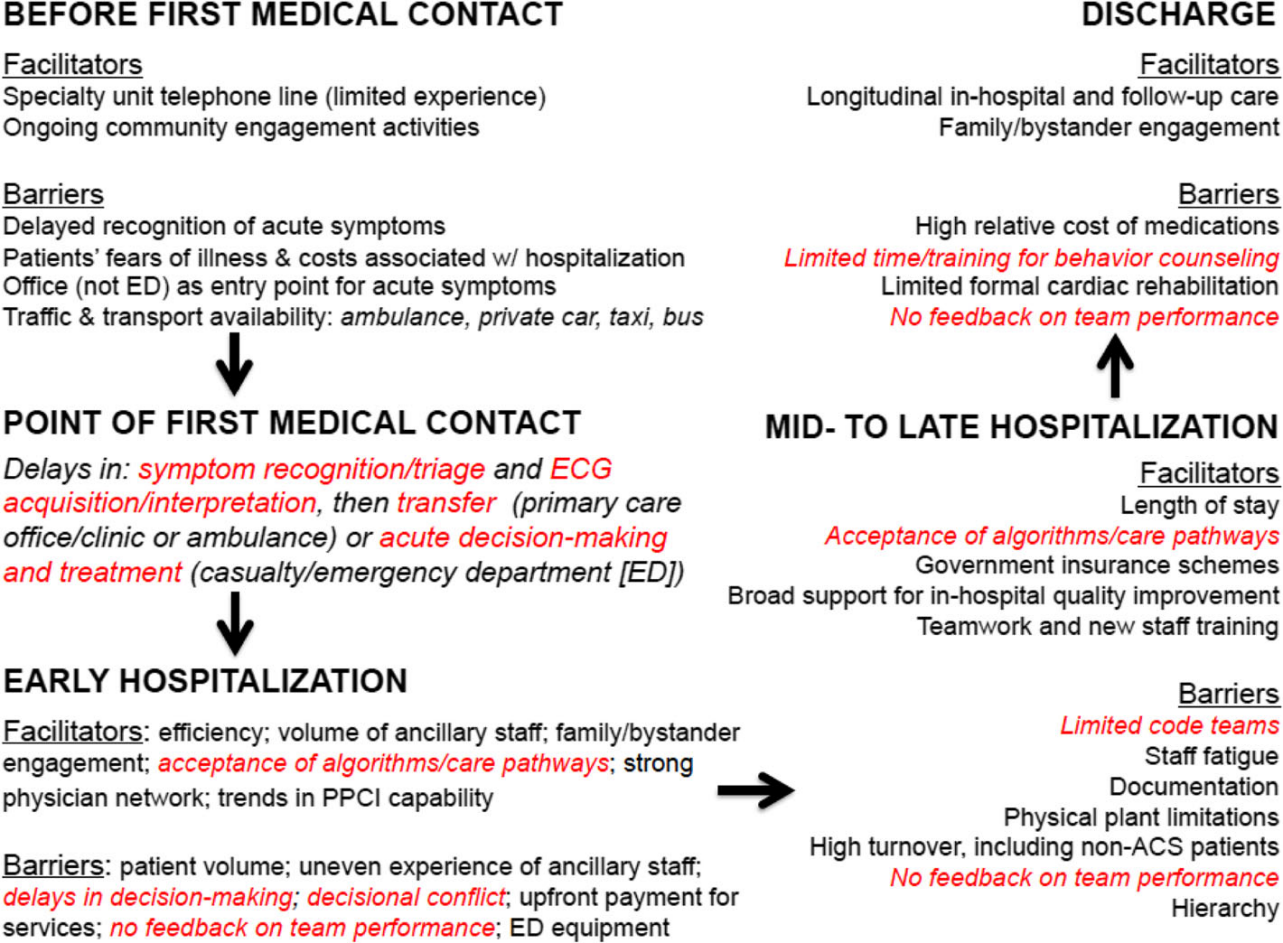
Critical care pathway used to develop the ACS QUIK trial quality improvement toolkit intervention. The barriers and facilitators were identified through in-depth interviews and focus group discussions with physicians/cardiologists to inform the development of the ACS QUIK trial quality improvement toolkit intervention. Phrases in red were potential targets of the toolkit intervention

To develop the ACS QUIK toolkit intervention, we conducted semi-structured interviews and focus group discussions with cardiologists and physicians who participated in the Kerala ACS Registry in English between May 2012 and November 2012. We purposely sampled and recruited 44 physicians or care providers (73% male; 43% cardiologist) using telephone and email invitations seeking a range of clinical experience and practice setting. Interviews and focus group discussions explored facilitators and barriers in the context of optimal ACS care in Kerala and were led by a cardiologist (MDH) and qualitative researchers (SG, DV). The audio recordings were transcribed, iteratively coded by two authors (MDH, KNK) until kappa > 0.8 to ensure agreement, and analyzed using Dedoose software v4.12 (Manhattan Beach, USA). The framework method [13] was used to create a contextualized critical pathway for patients with acute myocardial infarction with opportunities for intervention highlighted (Fig. 1).

We used high-quality systematic reviews [14–16] to inform adaption and implementation of previously reported strategies along with the results of the semi-structured interviews and focus group discussions to design the ACS QUIK toolkit intervention with the goal of improving processes of care and outcome measures [9]. The toolkit intervention included (1) a monthly audit and feedback reporting system based on key data elements used by the American Heart Association and American College of Cardiology [15, 17], (2) standardized admission and discharge order checklists [14], (3) translated and culturally adapted patient education materials related to tobacco cessation, dietary advice, and physical activity, and (4) access to free online quality improvement training through the Institute for Healthcare Improvement and linkage to emergency cardiovascular care training because of the low prevalence of code teams among the participating hospitals. These teams included code blue teams to respond to patients with cardiac arrest or respiratory failure and rapid response teams to treat patients who are acutely decompensated but have not developed cardiac arrest or respiratory failure. We also created telephone messaging groups via WhatsApp Messenger (WhatsApp, Inc., Mountain View, USA) within each cohort to facilitate communication among hospitals randomized to the same intervention step. We sent quarterly newsletters to all site investigators to provide general trial updates.

We generated monthly audit and feedback reports through the trial’s customized electronic data capture system (Data Template, Bangalore, India) with input from the research team for specifications and iterative testing. These reports were sent monthly via email to site investigators and included site-specific measures on performance, as well as hospital-level performance ratings compared to other hospitals in each cohort and all hospitals in the trial. A template of audit and feedback report is provided as online appendix.

#### ACS QUIK training at site initiation visits

In-person trainings were scheduled and conducted with all hospitals within 2 weeks prior to the scheduled crossover from the control period to the intervention period. Quality improvement teams were comprised of at least two hospital physicians, nurses, cardiac catheterization laboratory technicians, or staff who participated in acute myocardial infarction care, including cardiologists, emergency department physicians, and house officers. The 120- to 180-min training sessions were delivered in-person by a team of cardiologists and project coordinators from Northwestern University, USA, Centre for Chronic Disease Control (CCDC), India, and Westfort Hi-Tech Hospital Ltd., India. Training focused on strategies to decrease inappropriate thrombolysis, increase use of inexpensive essential cardiovascular medications, and minimize delays in selection of reperfusion strategy among patients with ST elevation myocardial infarction based on formative research (Additional file 1: Figure S1). Each hospital’s audit and feedback report was reviewed in detail by the trial and site teams with training related to organizing and leading monthly quality improvement team meetings. The goal of the training was to increase the use of evidence-based treatment and to provide sites with the training and flexibility to adapt and implement the toolkit intervention to maximize local utility. Additional training to new staff or refresher training to existing staff was provided by one of three zonal project coordinators during the study period who provided local training, monitoring, and support for the trial. Onsite monitoring visits and central statistical monitoring were performed during the trial to ensure data quality and completeness.

#### Post-toolkit intervention physician acceptability surveys and interviews

Surveys and interviews were performed at least 4 months after sites crossed over to the intervention period.

#### Online surveys

Between June 2015 and December 2016, online surveys were sent to all 63 hospitals’ investigators at least 4 months after the site had crossed over from the control period to the intervention period to explore perceived benefits and overall satisfaction after a learning period. The instrument captured (1) whether the participating hospital had established cardiovascular quality improvement teams, (2) frequency of quality improvement meetings, and (3) familiarity, use, and adaptation of the individual toolkit intervention components.

#### Physician interviews

From November 2015 to December 2016, one of three interviewers (KS, RD MDH) conducted 28 semi-structured in-depth interviews of physicians from 27 hospitals in English, either in-person or by telephone. The interviews were also performed at least 4 months after the site had crossed over into the intervention period. We used purposive sampling to ensure that we had a diversity of physicians based on the hospital size, cohort, and number of patients enrolled in the trial. The interviews aimed to collect information on what parts of the toolkit intervention were considered useful, the context in which the toolkit intervention was most easily used, perceived benefits, barriers to toolkit intervention implementation, and sustainability and scalability of the toolkit intervention (Additional file 2**:** Sample interview guide). The interview duration ranged from 10 to 30 min. The audio recordings were transcribed verbatim.

### Analysis

#### Survey data analysis

Online survey data were summarized using descriptive statistics. The mean and standard deviation are reported for continuous measures and frequency and percentages for categorical variables.

#### Interview data analysis

Using a thematic framework analysis approach [13], a codebook was developed through line-by-line reading of every interview transcript and categorization of transcript sections into emergent themes. Coding was completed by two authors (KS, RD) using qualitative data analysis software (MAXQDA, VERBI GmbH, Berlin, Germany). The descriptive codes were revised as grouped into prevailing themes, and data segments within each of the codes composing a given theme were thematically analyzed. Emerging categories were compared within and between respondents in an iterative process. The patterns identified in the analysis formed an analytical framework; thematic saturation of the emerging framework was reached as we found fewer differences arising in patterns.

Results of the thematic framework evaluation were set alongside results from the ACS QUIK trial to draw further inference about trial results. Physicians’ interview data, representing acceptability of toolkit intervention components (hospitals implementing all four toolkit intervention components were assigned a toolkit implementation score of 4, and those implementing any three, two, or one toolkit intervention components were assigned a score of 3, 2, and 1, respectively), were compared with changes in processes of care measures and clinical outcomes which occurred during the trial period. We performed an exploratory analysis of the trial’s primary outcome by restricting the sample to the 27 sites who participated in the process evaluation interviews. Mixed effect logistic regression was used to estimate the effect of the toolkit intervention on process measures and outcomes adjusted for cluster, temporal trends and high (three or four out of four toolkit intervention components) versus low or no (two or fewer toolkit intervention components) usage of the implementation toolkit intervention. For statistical analyses, Stata (version 15.0, Stata Corp), SAS (version 9.4, SAS Institute Inc.), and R (version 3.3.0, R Foundation) were used.

Using a thematic framework analysis, we summarized the established relationships between the toolkit intervention components, underlying context, mechanisms, and related trial outcomes to offer a middle range conceptual model that is likely generalizable and could be tested in future studies.

#### Ethical considerations

We received institutional ethics committee approval for the study from Northwestern University, Centre for Chronic Disease Control, participating hospitals, and the Cardiological Society of India-Kerala chapter (CSI-K) for hospitals without local ethics boards. All physicians provided written, informed consent prior to completing online survey and participating in in-depth interviews.

## Results

### Physician demographics

Of the 63 participating hospitals, 22 physician site investigators (35% response rate) completed the online survey. We also interviewed 28 physicians (44%) from 27 hospitals who were selected from different cohorts with different hospital size and patient recruitment. Physicians’ mean (SD) age was 52.5 (11.1) years, 96% were men, 84% had sub-specialty training in cardiology, and 33% worked at the government hospitals. Fourteen (45%) physicians who responded in the survey or interview were from small size hospitals (hospital size by patient recruitment < 200 patients), followed by 26% from medium (201–500 patients), 19% from large (501–1000 patients), and 10% from extra large (> 1000 patients) size hospitals.

### Online survey results

Table 1 reports the summary of online survey responses from trial physicians. Of the 22 site physicians who completed the survey, 17 (77%) reported that their hospital had a cardiovascular quality improvement team. ACS QUIK toolkit intervention components were used by the quality improvement team, including physicians, at each participating hospital. Most (*n* = 18, 82%) respondents had read an audit report, admission checklist, or discharge checklist and reported using patient education materials (*n* = 19, 86%). However, low implementation of establishing code blue team (32%) was reported.

**Table 1:** Physicians’ (respondents) characteristics and use of ACS QUIK toolkit intervention components: online survey results

Respondents characteristics	Total [*N* = 39]^a^
Mean age (in years) (SD)	52.5 (11.1)
Males, *N* (%)	38 (97)
Cardiology training (%)	84
Mean years of cardiology practice (SD)	12.8 (6.6)
Working at government hospitals (%)	37
Working at private hospitals (%)	63
Survey domains	Implementation/usage rate (%) *N* = 22
Established cardiovascular quality improvement team	77%
Viewed monthly audit and feedback report	82%
Used ACS QUIK admission checklist	82%
Used ACS QUIK discharge checklist	82%
Used ACS QUIK patient education materials (any of 3)	86%
Diet and lifestyle materials	86%
Tobacco cessation materials	83%
Cardiac rehabilitation materials	54%
Available training for the development of code or rapid response team	45%
Established code blue (cardiac arrest) team	32%
Established rapid response team	50%

*SD* standard deviation

^a^Inclusive of all physicians who participated in either online survey or in-depth interview

### Physician in-depth interview results and triangulation of ACS QUIK trial qualitative interview data

Analysis of physicians’ interview data revealed four major themes (utility of ACS QUIK toolkit intervention, adaptations to the existing toolkit intervention, sustainability, and recommendations for future use). Summary of key themes along with physicians’ quotes as supporting evidence are provided in Table 2.

**Table 2:** Qualitative themes, codes, and illustrative quotes (ACS QUIK toolkit intervention implementation facilitators and barriers are summarized separately in Tables 3 and 4 (below))

Themes	Codes	Illustrative quotations
Usefulness/acceptability of ACS QUIK toolkit intervention	a) Overall impressions of the toolkit intervention b) Audit and feedback report c) Admission and discharge checklists d) Patient education materials e) Guidelines to establish rapid response and code blue team	a) “(F)rom the QUIK kit (ACS QUIK toolkit intervention) point of view, the basic difference the ACS QUIK has done is that we made ourselves a little more efficient by way of transfer of patients from the ER (emergency room) to the ICU (intensive care unit) and the starting of treatment, antiplatelet and medicines.” [Site id: 19; toolkit intervention implementation score: 3] b) “(In the audit report), (w) e see our hospital stands in the 77th percentile, I am so happy to note that we are giving all the medicines that are required. Similarly, at the time of discharge, we go through the checklist and find that all essential medicines are given to the patient. We have given instruction that as soon as a patient comes with chest pain, ECG should be taken. Any sort of emergency, we shift them to the CCU (coronary care unit). We are routinely giving this one essential medicines. All the parameters mentioned, we are fairly doing it.” [Site id: 25; toolkit intervention implementation score: 4] c) “(D) efinitely (useful). Because even if everything said and done. Beta blockers, ACE-inhibitors, when discharge checklist come, some will be missing, because BP (blood pressure) will be borderline, so when we see the checklist we initiate it, so there is change in clinical practice.” [Site id: 18; toolkit intervention implementation score: 4] d) “We keep it (patient education material) on table, every time morning and evening shifts changes so whoever (doctors/nurses) is there uses that stuff (education materials for every patient coming to the cardiac care unit routinely)” [Site id: 4; toolkit intervention implementation score: 3] e) “No, we do not have a rapid response team in our hospital but we recently applied for NABH (National Accreditation Board for Hospitals), and it’s on the way and shortly we will be getting this approval … . and we will be forming code blue and rapid response team once we get approval from the NABH.” [Site id: 25; toolkit intervention implementation score: 4]
Adaptations to the ACS QUIK toolkit intervention	a) Admission and discharge checklists b) Patient education materials	f) “Other thing we change (referring to the discharge checklist) the medicine according to the other co-morbidities of the patients like bronchial asthma.” [Site id: 19; toolkit intervention implementation score: 3] g) “(S) o, we just made exercise and smoking cessation pictures from your material (ACS QUIK toolkit) and made sure that we ask them (junior doctors, nurses, or physiotherapist) to give 15 exercises and give them (patients) general instructions. We made a booklet. Most of our patients are not very educated, and we have some simple instructions on how they (ACS patients) can start walking, how long they can walk, what actions they should take if they (ACS patients) develop some symptoms. We have also given some algorithm also if patient develop some complications due to medications. Those things were framed in simple sentences. Also, for exercise and diet we made simple sentences.” [Site id: 2; toolkit intervention implementation score: 3]
Sustainability of ACS QUIK toolkit intervention	a) Use of toolkit intervention beyond trial period b) Sustainability of toolkit intervention components c) Barriers to toolkit intervention implementation: Lack of understanding of quality improvement program	h) “(W) hole heartedly (continued use of toolkit post-trial), as long as I am a cardiologist.” [Site id: 11; toolkit intervention implementation score: 3] i) “That (sustainability) is very important from the point of view of every person who is involved in patient care. It is not for the study alone. It is for the betterment of your patients.” [Site id: 49; toolkit intervention implementation score: 3] j) “Yes, it can be sustained but may not continue using the checklists beyond the trial period. I think the educational materials are most useful in long term.” [Site id: 19; toolkit intervention implementation score: 3] k) “All parts are useful, but the admission and discharge checklist is not very useful. Ours is a hospital run by a group of cardiologists. We have our own practice of prescribing medications at admission and discharge and therefore we do not look into the checklists provided by the study whether the medicines listed have been prescribed or not.” [Site id: 49; toolkit intervention implementation score: 3] l) “(M) aybe I will weigh that (audit report) as least useful of the four components, from patient management part, checklist certainly has utility, patient education material – yes. Audit report is only making us aware how we are faring in relation to other participating team/units. It is just a comparative data analysis. Nothing more comes out of it. Suddenly, you find that your hospital is at bottom, and then there is natural instinct to come somewhere in middle or top of the order.” [Site id: 19; toolkit intervention implementation score: 3] m) “I think basically it is the ignorance of the quality improvement program that is creating the issue. Or how to participate in a quality control program that is totally different from the normal trials (drug trials) that we are doing … So this is different and that’s where the problem (understanding the implementation of quality improvement trials) has come.” [Site id: 24; toolkit intervention implementation score: 3]
Recommendation to use toolkit intervention to other hospitals (Scale-up)	d) Tertiary care government and private hospitals e) Secondary care settings	n) “(Recommend to other hospitals)...100%, then only when others will use it, they (hospitals) will also understand the importance.” [Site id: 11; toolkit intervention implementation score: 3] o) “(Y) eah of course, that (use of toolkit components in long-run) will definitely change the pattern of the treatment … so I would strongly recommend that toolkit to be implemented in other hospitals also.” [Site id: 61; toolkit intervention implementation score: 0] p) “I think an ideal setting would be when it is used by non-cardiologist physicians.” [Site id: 49; toolkit intervention implementation score: 3]

### Utility of ACS QUIK toolkit intervention (by each component)

Most physicians (among 84% cardiologists) reported the toolkit intervention was useful, but few could implement all four components. Although the educational materials related to diet, activity, and tobacco cessation were generally reported to be the most useful, physicians felt that their scope for impact was reduced due to high patient volume and patients’ concomitant family needs. Checklists were perceived as the next most useful component by respondents. Hospitals that could not coordinate or deploy a cardiac arrest or rapid response team reported that this was due to lack of coordination or support from different departments. Some physicians reported that the toolkit intervention made them more alert and efficient during transitions of care.

### Audit and feedback report

Audit and feedback reports were sent via email to the hospitals’ site investigator every month but were reviewed by a minority of physicians (example report in the Appendix). Physicians expressed that, if the final study results demonstrated that a large proportion of patients were not prescribed optimal medical care, then an audit report might be helpful for physicians to investigate why these drugs were not prescribed. Physicians accepted that these reports added potential value in reviewing how their hospital performed compared with others in the study. When reviewing the audit report, physicians generally reviewed what proportion of patients received medical treatments and delays in treatment.

(In the audit report, we usually see) what (proportion of) patients are receiving the treatment, delay in treatment, we are concentrating more (to improve treatment practice), some aspects are difficult to change, we are actually trying to improve our resuscitation timing and improve our drug therapy. [Site ID: 2; Toolkit intervention implementation score: 3]

Hospitals that were more likely to review the audit and feedback reports were also more likely to understand their relative performance compared with their peer institutions.

Yeah definitely (useful), I just want to know where we stand in comparative (in the audit report). [Site ID: 15; Toolkit intervention implementation score: 4]

I think they (quality improvement team members) are absolutely happy we are able to be in the top quarter most of the time (in the audit report). [Site ID: 13; Toolkit intervention implementation score: 3]

The most common reasons mentioned by the physicians for not reviewing the audit report were time constraints, delegating responsibility to junior physicians, no patients enrolled in the study over the past 3 months, and slow Internet connectivity leading to a lag in data entry to make reporting contemporary. Physicians also expressed concern that their prescription rates for angiotensin-converting enzyme (ACE) inhibitors and angiotensin receptor blockers were generally low compared to global reports due to a perceived high prevalence of contraindications. Few physicians requested re-training of their hospital site staff to enable them to read, understand, and take corrective or proactive action by review of the audit reports.

### Admission and discharge checklists

All (*n* = 28) physicians reported that checklists were considered important for overall team performance. Some respondents reported using their own hospital-specific checklist (treatment protocol) or discharge summary template or adapting the checklist to suit their setting.

… most useful, discharge checklist because if something is missing, we are aware at discharge that we are aware and we check lot of things in that. [Site ID: 18; Toolkit intervention implementation score: 2]

Others reported that checklists made minimal difference in their clinical practice. Some physicians asserted that even in the absence of checklist, they closely followed clinical practice guidelines in prescribing optimal medical care. Checklists appeared to be more helpful for less-experienced physicians, including trainees.

### Patient education materials

Most physicians reported that patient education materials related to diet, physical activity, and tobacco cessation were displayed in the hospital and were routinely distributed and discussed with patients. Physicians reported that organized services for cardiac rehabilitation were not available at their site, which limited their use of cardiac rehabilitation materials that included home-based cardiac rehabilitation. Some hospitals employed a physiotherapist during the trial, but these service lines have yet to be fully developed for these patients in Kerala.

### Guidelines for development and deployment of code and rapid response teams

Most (*n* = 24, 86%) respondents reported that their hospitals had not yet operationalized a cardiac arrest or rapid response team.

### Toolkit intervention components that were less useful

Tertiary care hospitals that were staffed by senior cardiologists, residents, and physician assistants were less likely to find checklists useful. One physician criticized that there was no provision to add details on surgical procedures or angioplasty on the discharge checklists, which referred only to myocardial infarction or stroke.

### ACS QUIK toolkit intervention adaptations

There were no major suggestions to modify the current toolkit intervention components, other than additional local adaptations to discharge checklists and dietary and exercise education materials. For example, one site added details of the surgery or other intervention procedures performed in the discharge checklist. Few sites modified the patient education materials to include information on the starting dose and step-up dose of exercise post-discharge and dietary recommendations on salt intake. Of the 28 physicians interviewed, 29% (*n* = 8) reported either use of existing materials or local adaptations of the ACS QUIK toolkit intervention to contextualize its components. The patient education materials and discharge checklists were most commonly modified.

One physician reported implementation of other quality improvement measures during the study period, such as informal meetings in the department to review difficult cases, as well as random reviews of patient discharge summaries prepared by residents. Physicians also reported adaptation of the discharge checklist by expanding it to include medications for co-morbidities.

### Sustainability

All respondents agreed that hospitals should follow standard treatment protocols and guidelines for acute myocardial infarction treatment and management, including through the use of checklists. However, sites sought additional support from the coordinating center to help them implement the toolkit intervention components more consistently and reported that provision of educational materials would be the easiest component to sustain. Sites received payments for participation in the ACS QUIK trial (up to INR 750/US$12 per patient). Whether data collection and toolkit intervention implementation would continue without site payments remains uncertain. Some respondents identified the inherent nature of their clinical work as physicians as being an important driver for sustainability.

### Recommendation to other hospitals and continued use post-trial

Physicians supported the toolkit intervention concept and expressed interest in using the toolkit intervention component beyond the trial duration and were willing to recommend the toolkit intervention to other practitioners/hospitals, including in rural settings.

Those (hospitals) who are not following this, I think they are missing a point. Because when we see discharge summaries from some other hospitals or some other consultants, we see that there is deficiency from many aspects. So, going through the (check) list and somebody prompting them helps. [Site ID: 15; Toolkit intervention implementation score: 4]

Physicians had already shared the patient education materials that were given as part of the toolkit intervention to other consultants in a neighboring hospital.

(D) efinitely (recommend to other hospitals). In fact, I gave some of your material to neighboring hospital who is practicing good cardiology. [Site ID: 5; Toolkit intervention implementation score: 2]

### Facilitators and barriers to implementation of toolkit intervention

**Fig. 2:**
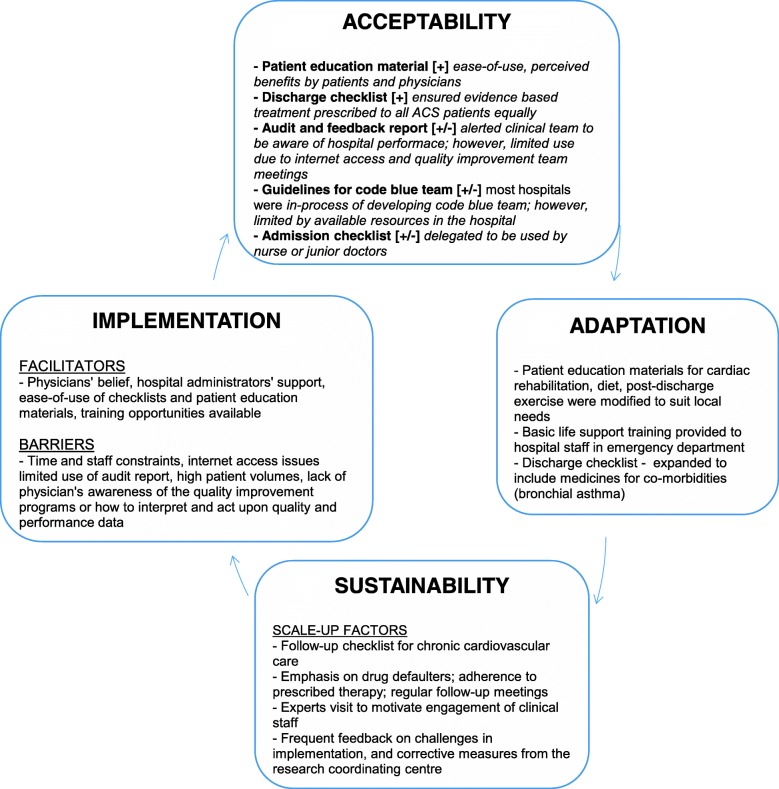
Conceptual model to inform the factors influencing the implementation, acceptability, and sustainability of the ACS QUIK toolkit intervention. This figure describes the conceptual model to inform the factors influencing ACS QUIK toolkit intervention implementation, acceptability by study physicians, and sustainability of scale-up factors

Tables 3 and 4 demonstrate the barriers and facilitators to ACS QUIK toolkit intervention implementation following synthesis of survey and qualitative interview data. Physicians’ belief and support from hospital administrators, usefulness and ease-of-use of checklists, and trial patient satisfaction with the care provided by the cardiovascular quality improvement team were the main facilitators identified. Hospitals that could not implement the toolkit intervention components reported problems such as change in hospital staff who were previously handling the program implementation and Internet connectivity problems. Figure 2 displays the conceptual framework for implementation, acceptability, adaptation, and sustainability of the ACS QUIK toolkit intervention.

**Table 3:** Facilitators to the implementation of the ACS QUIK toolkit intervention

Facilitators	Data source	Description	Context, conditions, and consequences
Individual level			
Physicians believed in the toolkit intervention	Interview	Physicians’ engagement was a function of initial views about ACS QUIK toolkit intervention	Physicians’ engagement in implementing the toolkit intervention was shaped by their interest with awareness and initial belief in the toolkit intervention that it will be beneficial to improve patient outcomes.
Usefulness of checklists and patient education materials	Survey, interview	Admission and discharge checklists and patient education materials were simple and easy to use	In view of high patient volume and physicians’ time constraints, admission and discharge checklists were easy to administer and patient education materials were distributed to patients and their relatives in the outpatient clinic or at the discharge visit.
Patients satisfaction with the care provided by the cardiovascular quality improvement team	Survey, interview	Patients responded positively to the care provided by the cardiovascular quality improvement team.	Physicians expressed that patients liked the education materials and care provided by the ACS QUIK trial team.
Organizational level			
Inter-departmental communication	Interview	Coordination between medicine department, coronary care unit, and emergency unit department was influenced by the implementation of toolkit intervention	Involvement of physicians, consultants and support staff from various departments viz. emergency unit, coronary care unit, and medicine department improved transfer communication and better delivery of toolkit intervention.
Training opportunities available to form code /rapid response team	Survey, interview	Code (cardiac arrest) team and rapid response teams were established after training guidelines were provided to the hospitals.	Training opportunities were made available to the hospital teams to create code and rapid response team to improve resuscitation procedures, door-to-needle or door-to-balloon time, and ultimately patient outcomes.
Organizational support	Interview	Support of the hospital administrators	Hospital administrators and physicians supported the view of delivering standardized treatment protocol to all ACS patients.

**Table 4:** Barriers to the implementation of the ACS QUIK toolkit intervention

Barriers	Data source	Description	Context, conditions, and consequences
Individual level			
Time and staffing constraints (frequent change in hospital nursing staff)	Interview	Limited nursing staff and physicians available at the hospital and frequent change in nursing staff	Limited staff availability and frequent turnover in nursing staff affected continuity in delivering the toolkit intervention components and hence, its overall effect on patient outcomes could have been affected.
Inadequate understanding of the quality improvement programs	Interview	Physicians did not fully understand the difference between drug trials and quality improvement translation trials.	Because of lack of physicians’ awareness of quality improvement programs, the approach to the implementation of toolkit intervention suffered and there was inadequate response to audit reports.
Organizational level			
Technological constraint: electronic case report forms and audit report relying on internet access	Interview	Access to uninterrupted internet services was not available at all hospitals	Due to slow internet access, there were lags in data entry and in accessing audit reports.
High patient volumes and lack of inter-department coordination/ communication	Interview	High patient volumes and lack of coordination/communication between emergency unit, medicine department, and coronary care unit	Due to high patient volumes, physicians or support staff could not explain the patient educations materials including tobacco cessation, cardiac rehabilitation in as much detail as possible. Also, lack of coordination/support from various departments hindered the full implementation and delivery of toolkit intervention components, including changes to the clinical flow of patients with acute myocardial infarction.
Lack of adequate training to the support staff	Survey	Physicians expressed that additional training to support staff could lead to improved delivery of toolkit intervention.	Additional training to support staff on regular intervals was sought and received by the site physicians to improve delivery of toolkit intervention to account for frequent turnover of support staff.
Low patient enrolment rates	Interview	Few hospitals could not use audit reports in meaningful ways due to low patient enrolment rates, including low consent rates.	Since the audit report summarized the hospital-level performance and measures based on patient data entered in the system, sites that enrolled few patients did not have meaningful indicators in the audit report.

### Integrating results of the process evaluation with ACS QUIK trial outcomes

Among ACS QUIK patients (*n* = 12,686) enrolled by the 27 hospitals that participated in this mixed methods implementation evaluation, patients in the intervention phase were older, had a higher body weight, had higher fasting glucose and prevalence of diabetes, had lower rates of tobacco use and health insurance, were less frequently transferred from a different hospital, and had lower troponin, LDL cholesterol, creatinine, and hemoglobin compared to patients in the control phase (Additional file 3: Table S1). Sites that participated in the process evaluation, demonstrated lower major adverse cardiovascular event (MACE) rates in the intervention phase compared to the control phase, although the effects were attenuated after adjustment for cluster, temporal trends, and degree of toolkit intervention implementation (Additional file 4: Figure S2 and Additional file 3: Table S2). Sites that implemented 3 or 4 out of 4 toolkit intervention components demonstrated higher rates of process measures in the intervention group compared to control (Additional file 4: Figure S2 and Additional file 3: Table S3), and sites that implemented two or fewer toolkit intervention components had similar rates between the intervention and control groups (Additional file 4: Figure S2 and Additional file 3: Table S4).

Table 5 demonstrates the toolkit intervention, contextual, and mechanistic factors that could have influenced the study outcomes, which are further categorized as either observed, implied, or anticipated based on this mixed methods evaluation. The key findings observed were improvements in processes of care measures, such as reperfusion among patients with ST-segment elevation myocardial infarction and prescription of in-hospital and discharge cardiovascular medications (aspirin, statins, and blood pressure-lowering agents) associated with the use of admission and discharge checklists. Since the ACS QUIK trial results were unknown during the conduct of process evaluation, we have only hypothesized contextual and underlying mechanisms, which could be tested in future studies.

**Table 5:** Synthesis of quantitative and qualitative data to inform intervention factors, context, and underlying mechanisms influencing outcomes

Intervention components	Level intervention is operating	Intervention factors	Contextual factors	Mechanistic factors	Outcome	Outcome^a^: observed, implied, or anticipated
Audit and feedback report	Hospital/institution	Audit report summarized the key performance indicators of hospital in comparison to other hospitals in the cohort and all the hospitals participating in the ACS QUIK trial.	Formative work identified lack of systems to track quality of care indicators	We hypothesize that monthly review of audit report stimulates clinical team to set goals to make changes that will improve processes and clinical outcomes.	Clinical team used evidence provided in the audit report for 50–85% of their patients but quality improvement meetings were rare and changes in clinical practice based on these data were not identified.	Observed
Admission and discharge checklists	Physician, nurse	Admission and discharge checklists incorporated evidence-based guideline recommended treatment for acute myocardial infraction care at admission and discharge.	To minimize variability in practice across hospitals and promote checklists to embed evidence in decision-making	Checklists enhance prescription of evidence-based treatment in-hospital and at discharge.	Overall improved prescription of aspirin, beta-blocker and statins at discharge.	Observed
Patient education materials	Patient	Patient education materials were developed with a focus on tobacco cessation, diet, exercise and cardiac rehabilitation post-acute myocardial infarction.	Lack of tobacco cessation counseling, heart-healthy diet and exercise information for patients with acute myocardial infarction	Patient education sped-up recovery post-event and reduce the risk of recurrent event.	Education material may have enhanced patient self-care post-acute myocardial infarction.	Implied
Guidelines to develop rapid response and code blue team	Hospital/institutional	Guidelines and relevant training were provided to the team to establish rapid response and code teams.	Absence of rapid response and code team in most settings	Development of rapid response and code team facilitated clinical team to do their work more efficiently and are therefore valued.	Evidence will be considered more systematically across departments when policy is developed and implemented.	Anticipated

^a^Outcome definition: observed (directly evident from the data), implied (no direct data available but interpreted based on triangulated results), or anticipated (based on assumptions guided by the interview or survey data)

## Discussion

### Summary of results

This mixed methods evaluation of the ACS QUIK trial processes sought to describe the contextual development of the toolkit intervention, evaluate experiences and perceptions of physicians while implementing the trial, and explore facilitators, barriers, and context for implementation, acceptability, adaptation, and sustainability of the toolkit intervention. Our findings suggest that the presence of cardiovascular quality improvement teams, regular review of audit and feedback reports, checklists, regular quality improvement meetings and wider dissemination of patient education materials resulted in improved processes of care. However, a relatively high level of care at baseline in the control group hospitals (e.g., > 95% rate of discharge aspirin use), incomplete toolkit intervention implementation with a corresponding modest effect on process of care measures, and favorable temporal trends (i.e., background improvements in clinical care over the study period) limited the effect of the toolkit intervention on clinical outcomes.

### Explanation of results

This mixed methods implementation evaluation of the ACS QUIK trial provides insights into the interaction between the toolkit intervention and its external context. We identified themes related to the quality improvement toolkit intervention’s integration of new activities into existing activities that changed behavior and performance through regular review. These findings may help explain both how the toolkit intervention operated and the mixed results of the trial. Mixed methods evaluation based on Medical Research Council’s framework provided a useful theoretical framework for this process evaluation by allowing a specific focus on context. Although complex interventions might appear “out of control” due to their varied manifestation in different situations, context-sensitive process evaluations can help identify interventions’ key functions.

Our findings also indicate the need for concurrent investment in improving the structural capacity of hospitals to achieve optimal outcomes [18]. The responsiveness of the pre-hospital environment [19, 20] and capacity of hospitals' emergency cardiovascular care services, essential structural prerequisites to deliver optimal acute myocardial infarction care, varied considerably among ACS QUIK trial hospitals. Lack of standardization of emergency cardiovascular care, limited funding, and insufficient numbers of qualified emergency staff have been identified as contributing to such variation. Similarly, systems and resources to support quality improvement varied across participating hospitals, including existing data capture systems, dedicated quality officers, effective management, and buy-in among hospital leadership. Future quality improvement initiatives will need to address these structural limitations without which substantial improvement in care is unlikely to be achieved.

### Results in context

Our study provides unique data on the implementation factors, acceptability, adaptation, and sustainability of an acute myocardial infarction quality improvement toolkit intervention within a low- and middle-income country context. The mixed methods evaluation explored the context and the extent of adherence to the delivery of the ACS QUIK toolkit intervention and trial outcomes, the results of which suggest that higher implementers (three or four toolkit intervention components) had higher rates of process of care measure improvements but similar rates of the study’s primary outcome. These variations could be driven by differences in what was implemented (type of toolkit intervention component) and to what degree that led to meaningful change in outcomes, as well as differences in the sites (hospital size, resources), their teams, patients, baseline process of care and major adverse cardiovascular event rates, the way in which sites implemented the toolkit intervention (or not), or chance.

One study from Brazil reported improvements in medication use and reperfusion among patients with ST elevation myocardial infarction with increased use of evidence-based treatment [3]. However, this study did not evaluate the acceptability of the toolkit intervention from various stakeholders (providers, patients, or hospital administrators) perspective including physicians, patients, or hospital administrators. Another quality improvement trial among ACS patients in China (CPACS-2) performed qualitative evaluation and found that system-level barriers affected the ability of clinical pathways to change practice [4, 5]. A similarly designed trial in China that is powered to detect a potential difference in clinical outcomes has been completed and may help place results from ACS QUIK into broader context [21]. Our results are consistent with these findings in highlighting the role of hospital administrators and leadership support for implementing quality improvement toolkit interventions. However, further research is required to understand how to evaluate and improve hospital management and leadership support and performance in low- and middle-income countries [22].

### Strengths and limitations

Our study has several strengths. First, our mixed methods evaluation was built upon our previous observational study from Kerala ACS Registry and was executed in partnership with the Cardiological Society of India-Kerala Chapter. Second, we captured views and experiences of a range of settings (government, private, and nonprofit hospitals) prior to unblinding of the results and have created a model that is likely generalizable and could be tested in future studies. We used exploratory interviews with physicians to assess the context of implementing the ACS QUIK toolkit intervention prior to unblinding the ACS QUIK trial results. Therefore, our process evaluation collected information about trial context and mechanisms within those contexts at the time of the trial with neither the researcher nor the physicians being influenced by the trial results. Third, this mixed methods approach facilitated systematic examination of the empirical trial data along with interview data to build and adapt a middle-range conceptual model. The emergent theory helped us to develop an understanding about how and why the toolkit intervention was developed, incorporating the interaction between context and mechanisms, explaining the key functions of the toolkit intervention and how this translated to the study outcomes.

Our study also has important limitations. Low participation rates in the online survey to assess acceptability of toolkit intervention provide a limited view on the overall acceptance rates and strategies to further improve toolkit intervention implementation. The process evaluation may also have been subject to social desirability bias among trial physicians. Measures were taken to reduce this likelihood, including (1) assuring confidentially and anonymity, (2) assuring respondents that their candid views would help towards improving the intervention in the future, and (3) their candor was critical for the larger cause of improving cardiovascular quality and safety in Kerala. Another limitation of our process evaluation was restricted to physicians, which is relevant since other healthcare workers may influence acute cardiovascular care quality and safety. Also, we did not have the trial results when undertaking the interviews, which may have limited the ability to probe for specific results and even response rates. Follow-up interviews with physicians after knowing the trial outcomes might have led to investigating specific drivers for neutral results and to exploring the factors that have influenced the study results. On the other hand, results may have biased physicians’ perceptions. Lastly, in our post hoc quantitative analyses, there could be potential unmeasured confounding between sites, site investigators, toolkit intervention implementation, and clinical outcomes, which are difficult to quantify or control.

## Conclusions

Implementation and acceptability of ACS QUIK toolkit intervention was enhanced by the hospital-level management support, clinical team enthusiasm, and ease of using checklists and patient education materials but was limited by time and staff constraints and understanding of quality improvement programs. Wider use of a similar toolkit intervention to other acute or chronic cardiovascular conditions (e.g., heart failure and stroke) or other locations where the quality of care is not as high as in the ACS QUIK trial may be useful for improving acute cardiovascular care in India and other low- and middle-income country settings.

## Additional files

Additional file 1: **Figure S1.** Reperfusion decision-making framework in patients with ST-segment elevation myocardial infarction in Kerala. (DOCX 2569 kb)

Additional file 2: Sample interview guide. ACS QUIK Process Evaluation Interview Guide (DOCX 18 kb)

Additional file 3: **Table S1.** Baseline characteristics in ACS QUIK patients by intervention and control group among 12,688 patients enrolled by 27 hospitals participating in the process evaluation interviews. **Table S2.** Unadjusted and adjusted primary and secondary trial outcomes using mixed effect logistic regression models that account for within-hospital clustering and clustering, temporal trends and implementation of the toolkit intervention. **Table S3.** Unadjusted and adjusted primary and secondary trial outcomes using mixed effect logistic regression models that account for within-hospital clustering and clustering and temporal trends among sites that implemented three or four ACS QUIK toolkit intervention components. **Table S4.** Unadjusted and adjusted primary and secondary trial outcomes using mixed effect logistic regression models that account for within-hospital clustering and clustering and temporal trends among sites that implemented two or fewer ACS QUIK toolkit intervention components. (DOCX 45 kb)

Additional file 4: **Figure S2.** Rate of major adverse cardiovascular events at 30 days in the intervention and control groups within the ACS QUIK trial, as well as adjusted difference and adjusted odds ratio (95% confidence intervals) stratified by sites by participation in process evaluation interviews and number of quality improvement toolkit intervention components implemented (DOCX 111 kb)
