# Supplementary material for: Implementation and acceptability of a heart attack quality improvement intervention in India: a mixed methods analysis of the ACS QUIK trial
Source: Implement Sci. 2019 Feb 6;14:12. doi: 10.1186/s13012-019-0857-7 (PMC6364470; doi:10.1186/s13012-019-0857-7)
Supplement: Supplementary file 1 — Figure S1. Reperfusion decision-making framework in patients with ST-segment elevation myocardial infarction in Kerala. (DOCX 2569 kb) [file 13012_2019_857_MOESM1_ESM.docx]

Additional file 1: Figure S1**.** Reperfusion decision-making framework in patients with ST-segment elevation myocardial infarction in Kerala.

**
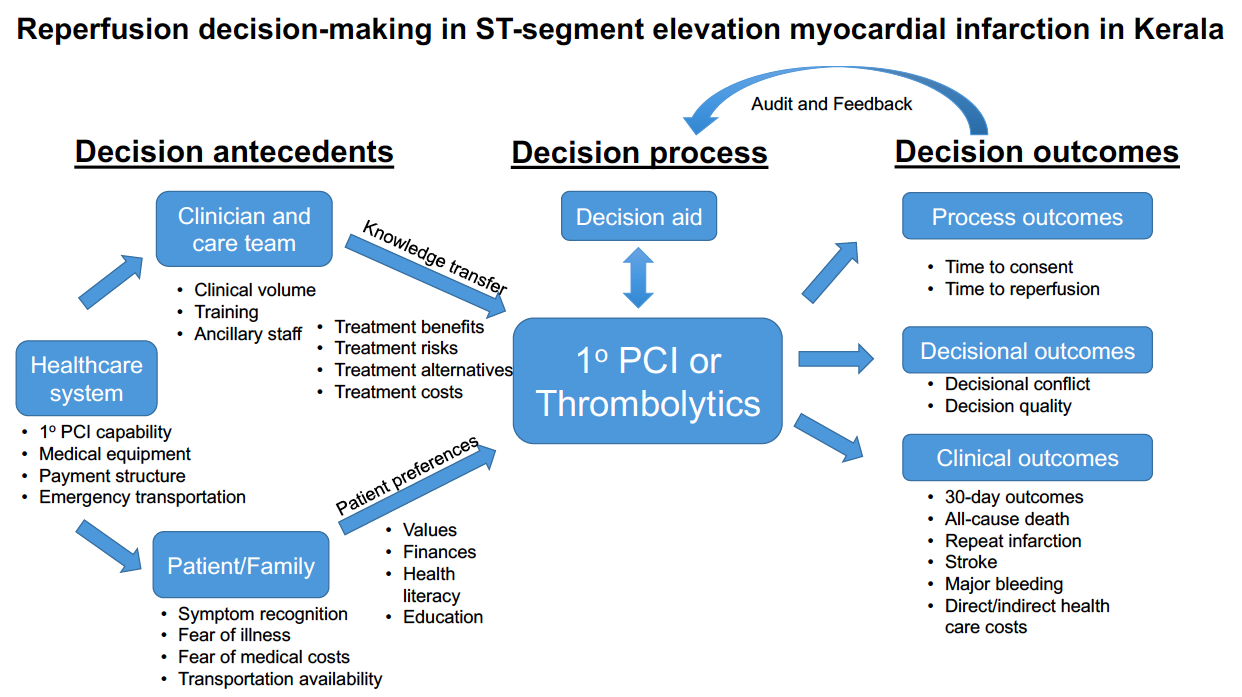
**

PCI=primary percutaneous coronary intervention.
