# Supplementary material for: Implementation and acceptability of a heart attack quality improvement intervention in India: a mixed methods analysis of the ACS QUIK trial
Source: Implement Sci. 2019 Feb 6;14:12. doi: 10.1186/s13012-019-0857-7 (PMC6364470; doi:10.1186/s13012-019-0857-7)
Supplement: Supplementary file 2 — Sample interview guide. ACS QUIK Process Evaluation Interview Guide (DOCX 18 kb) [file 13012_2019_857_MOESM2_ESM.docx]

Additional file 2

**Data Supplement: ACS QUIK Process Evaluation Interview Guide**

*v17 October 2015*

**Overview**

Interviews can be conducted with principal investigators, and quality improvement team members who understand what components of the quality improvement toolkit are being implemented.

Interviewees must have signed an informed consent, and must have been in the intervention phase of ACS QUIK for 4 months or more.

Interviews may be:

1. Face-to-face
2. Telephonic

**Draft principal investigator/quality improvement team members interview questions**

1. Have you completed the online survey?

If Yes, proceed to question 2

If No, ask for responses to the survey questions now

1. When was your hospital randomized to the intervention stage?
2. Tell me about the last time you used any component of the Acute Coronary Syndrome Quality Improvement in Kerala (ACS QUIK) toolkit.
3. Describe what part of the ACS QUIK toolkit was useful.
4. Describe what part of the ACS QUIK toolkit was not useful.
5. Let’s walk through the audit report together. Describe what components you find useful. What do you typically look at (if anything)?
6. Describe what should ideally be included in the ACS QUIK toolkit.
7. Describe the context when the ACS QUIK toolkit is most easily used.
8. Describe how your clinical practice has changed in the past four months, after being involved in the intervention.
9. Can the intervention be easily sustained? Which aspects of the intervention can be most sustainable in long term?
10. Will you consider recommending the trial intervention package to other clinic or hospital settings or like to continue with the intervention strategy after trial completion?
