# Supplementary material for: Implementation and acceptability of a heart attack quality improvement intervention in India: a mixed methods analysis of the ACS QUIK trial
Source: Implement Sci. 2019 Feb 6;14:12. doi: 10.1186/s13012-019-0857-7 (PMC6364470; doi:10.1186/s13012-019-0857-7)
Supplement: Supplementary file 3 — Table S1. Baseline characteristics in ACS QUIK patients by intervention and control group among 12,688 patients enrolled by 27 hospitals participating in the process evaluation interviews. Table S2. Unadjusted and adjusted primary and secondary trial outcomes using mixed effect logistic regression models that account for within-hospital clustering and clustering, temporal trends and implementation of the toolkit intervention. Table S3. Unadjusted and adjusted primary and secondary trial outcomes using mixed effect logistic regression models that account for within-hospital clustering and clustering and temporal trends among sites that implemented three or four ACS QUIK toolkit intervention components. Table S4. Unadjusted and adjusted primary and secondary trial outcomes using mixed effect logistic regression models that account for within-hospital clustering and clustering and temporal trends among sites that implemented two or fewer ACS QUIK toolkit intervention components. (DOCX 45 kb) [file 13012_2019_857_MOESM3_ESM.docx]

Additional file 3:Table S1**.** Baseline characteristics in ACS QUIK participants by intervention and control group among 12,688 participants enrolled by 27 hospitals participating in the process evaluation interviews.

| **Characteristics** | **Control**  **n=5032** | **Intervention**  **n=7656** | **Difference (95% CI)^a^** |
| --- | --- | --- | --- |
| Age, mean (SD), years | 60.4 ± 12.1 | 61.3 ± 12.2 | 0.83 (0.40 to 1.27) |
| Male, n (%) | 3795 (75.4) | 5742 (75.0) | -0.42 (-1.95 to 1.12) |
| History of tobacco use, n (%) | 1997 (39.7) | 1848 (24.1) | -15.55 (-17.21 to -13.89) |
| History of diabetes, n (%) | 2089 (41.5) | 3648 (47.6) | 6.13 (4.37 to 7.90) |
| Transferred, n (%) | 2327 (46.2) | 3180 (41.5) | -4.71 (-6.47 to -2.94) |
| No insurance, n (%) | 3599 (71.5) | 5723 (74.8) | 3.23 (1.65 to 4.81) |
| ST elevation myocardial infarction, n (%) | 3136 (62.3) | 4182 (54.6) | -7.70 (-9.44 to -5.95) |
| Symptom-to-door time, median (IQR), min | 270 (134-956) | 305 (125-915) | 35 (17 to 53) |
| Body weight, mean (SD), kg | 62.7 ± 9.4 | 63.1 ± 9.7 | 0.45 (0.10 to 0.79) |
| Systolic blood pressure, mean (SD), mmHg | 139.0 ± 29.9 | 139.9 ± 29.5 | 0.88 (-0.17 to 1.94) |
| Heart rate, mean (SD), bpm | 80.5 ± 19.3 | 80.3 ± 19.0 | -0.21 (-0.89 to 0.47) |
| Initial troponin, median (IQR), ng/ml | 1.6 (0.4-8.9) | 1.0 (0.2-4.0) | -0.66 (-0.85 to -0.47) |
| LDL cholesterol, mean (SD), mg/dl | 125.2 ± 40.2 | 116.0 ± 41.4 | -9.22 (-10.99 to -7.46) |
| Triglycerides, median (IQR), mg/dl | 128 (94-177) | 126 (93-167) | -2 (-5 to 1) |
| Serum creatinine, median (IQR), mg/dl | 1.1 (0.9-1.3) | 1.0 (0.9-1.3) | -0.1 (-0.1 to -0.1) |
| Fasting glucose, median (IQR), mg/dl | 120 (91-172) | 131 (105-180) | 11 (8 to 14) |
| Hemoglobin, mean (SD), mg/dl | 13.2 ± 2.1 | 13.1 ± 2.0 | -0.09 (-0.17 to -0.02) |
| Hospital type, n (%) |  |  |  |
| Government (n=7) | 2733 (54.3) | 2502 (32.7) | -21.63 (-23.36 to -19.90) |
| Non-profit/Charity (n=6) | 1410 (28.0) | 2062 (26.9) | -1.09 (-2.68 to 0.50) |
| Private (n=14) | 889 (17.7) | 3092 (40.4) | 22.72 (21.20 to 24.24) |
| Hospital size by anticipated enrollment, n (%) |  |  |  |
| Extra large (n=3; >1000) | 489 (9.7) | 1173 (15.3) | 5.60 (4.45 to 6.75) |
| Large (n=7; 501-1000) | 1263 (25.1) | 3065 (40.0) | 14.93 (13.31 to 16.56) |
| Medium (n=12; 201-500) | 3033 (60.3) | 2876 (37.6) | -22.71 (-24.44 to -20.98) |
| Small (n=5; ≤200) | 247 (4.9) | 542 (7.1) | 2.17 (1.34 to 3.00) |
| Catheterization lab, n (%) |  |  |  |
| Installed during study (n=2) | 29 (0.6) | 325 (4.2) | 3.67 (3.17 to 4.17) |
| No (n=6) | 1582 (31.4) | 1036 (13.5) | -17.91 (-19.40 to -16.41) |
| Yes (n=19) | 3421 (68.0) | 6295 (82.2) | 14.24 (12.69 to 15.79) |

Abbreviations: SD, standard deviation; IQR, interquartile range

^a^Crude difference = intervention minus control

Table S2**.** Unadjusted and adjusted primary and secondary trial outcomes using mixed effect logistic regression models that account for within-hospital clustering and clustering, temporal trends and implementation of the intervention toolkit.

| **Outcome** | **Control**  **(N=5032)** | | **Intervention**  **(N=7656)** | | **Cluster Adjusted Difference,**  **% (95% CI)**^a^ | **Cluster Adjusted**  **OR (95% CI)**^a^ | **Primary Analysis Difference,**  **% (95% CI)**^a^ | **Primary Analysis**  **OR (95% CI)**^a^ |
| --- | --- | --- | --- | --- | --- | --- | --- | --- |
|  | **n** | **%** | **n** | **%** |  |  |  |  |
| *Primary Outcome* |  |  |  |  |  |  |  |  |
| 30-day MACE | 376 | 7.5 | 402 | 5.3 | -1.03 (-2.03 to -0.03) | 0.84 (0.72 to 0.99) | -1.10 (-2.84 to 0.64) | 0.83 (0.63 to 1.10) |
| *Secondary Outcomes* |  |  |  |  |  |  |  |  |
| 30-day mortality | 301 | 6.0 | 298 | 3.9 | -0.96 (-1.86 to -0.06) | 0.81 (0.68 to 0.97) | -1.10 (-2.66 to 0.45) | 0.79 (0.58 to 1.08) |
| 30-day cardiovascular mortality | 295 | 5.9 | 290 | 3.8 | -0.92 (-1.80 to -0.04) | 0.81 (0.68 to 0.98) | -1.04 (-2.56 to 0.48) | 0.79 (0.58 to 1.09) |
| In-hospital mortality | 185 | 3.7 | 209 | 2.7 | -0.33 (-1.04 to 0.37) | 0.90 (0.72 to 1.12) | -1.46 (-2.97 to 0.05) | 0.64 (0.43 to 0.95) |
| 30-day re-infarction | 75 | 1.5 | 84 | 1.1 | -0.23 (-0.76 to 0.30) | 0.86 (0.62 to 1.20) | 0.16 (-0.90 to 1.21) | 1.11 (0.54 to 2.29) |
| 30-day stroke | 31 | 0.6 | 65 | 0.8 | 0.29 (-0.09 to 0.66) | 1.45 (0.90 to 2.32) | 0.22 (-0.39 to 0.83) | 1.32 (0.60 to 2.93) |
| 30-day major GUSTO bleeding^b^ | 8 | 0.2 | 21 | 0.3 | 0.17 (-0.07 to 0.40) | 1.82 (0.78 to 4.26) | 0.10 (-0.29 to 0.49) | 1.40 (0.34 to 5.75) |
| Optimal in-hospital medication^c^ | 1405 | 28.9 | 2900 | 39.6 | 8.46 (6.32 to 10.60) | 1.54 (1.39 to 1.71) | 11.38 (8.08 to 14.67) | 1.79 (1.52 to 2.11) |
| Optimal discharge medication^d^ | 2315 | 55.6 | 4542 | 66.5 | 10.94 (8.72 to 13.15) | 1.78 (1.61 to 1.96) | 11.37 (7.97 to 14.77) | 1.82 (1.54 to 2.14) |
| Tobacco cessation advice^e^ | 1872 | 95.9 | 1706 | 94.6 | 0.26 (-1.72 to 2.24) | 1.04 (0.75 to 1.46) | -0.83 (-4.57 to 2.92) | 0.87 (0.45 to 1.67) |

Abbreviations: MACE: major adverse cardiovascular events defined as death, reinfarction, stroke, and major GUSTO bleeding.

^a^Odds ratios represent effect of intervention compared with control and are calculated as the difference in marginal effects (intervention group minus control group) in a mixed-effects logistic regression model including a random-effects term to account for within-hospital clustering. Primary analysis additional accounted for temporal trends.

^b^Major bleeding is defined by the Global Utilization of Streptokinase and Tissue Plasminogen Activator for Occluded Coronary Arteries (GUSTO) criteria, which is defined by intracerebral haemorrhage or bleeding resulting in substantial hemodynamic compromise requiring treatment.

^c^Composed of aspirin, adenosine diphosphate receptor antagonist (clopidogrel, prasugrel, or ticagrelor), anticoagulant, and β-blocker among patients eligible to receive all medications.

^d^Composed of aspirin, adenosine diphosphate receptor antagonist (clopidogrel, prasugrel, or ticagrelor), statin, and β-blocker among discharged patients eligible to receive all medications.

^e^Among discharged patients who reported smoking at baseline.

Table S3**.** Unadjusted and adjusted primary and secondary trial outcomes using mixed effect logistic regression models that account for within-hospital clustering and clustering and temporal trends **among sites that implemented 3 or 4 ACS QUIK toolkit intervention components.**

| **Outcome** | **Control**  **(N=3673)** | | **Intervention**  **(N=4665)** | | **Cluster Adjusted Difference,**  **% (95% CI)**^a^ | **Cluster Adjusted**  **OR (95% CI)**^a^ | **Primary Analysis Difference,**  **% (95% CI)**^a^ | **Primary Analysis**  **OR (95% CI)**^a^ |
| --- | --- | --- | --- | --- | --- | --- | --- | --- |
|  | **n** | **%** | **n** | **%** |  |  |  |  |
| *Primary Outcome* |  |  |  |  |  |  |  |  |
| 30-day MACE | 244 | 6.6 | 212 | 4.5 | -0.44 (-1.69 to 0.81) | 0.93 (0.75 to 1.15) | -0.87 (-3.03 to 1.30) | 0.86 (0.61 to 1.23) |
| *Secondary Outcomes* |  |  |  |  |  |  |  |  |
| 30-day mortality | 206 | 5.6 | 153 | 3.3 | -0.82 (-1.98 to 0.35) | 0.84 (0.66 to 1.06) | -1.72 (-3.84 to 0.40) | 0.70 (0.47 to 1.04) |
| 30-day cardiovascular mortality | 201 | 5.5 | 149 | 3.2 | -0.75 (-1.88 to 0.39) | 0.85 (0.67 to 1.08) | -1.73 (-3.84 to 0.37) | 0.69 (0.46 to 1.03) |
| In-hospital mortality | 118 | 3.2 | 103 | 2.2 | -0.12 (-1.04 to 0.79) | 0.96 (0.72 to 1.29) | -1.50 (-3.48 to 0.47) | 0.63 (0.38 to 1.05) |
| 30-day re-infarction | 41 | 1.1 | 43 | 0.9 | -0.06 (-0.79 to 0.66) | 0.96 (0.60 to 1.53) | 0.25 (-1.07 to 1.56) | 1.18 (0.49 to 2.85) |
| 30-day stroke | 25 | 0.7 | 42 | 0.9 | 0.36 (-0.22 to 0.95) | 1.44 (0.83 to 2.50) | 0.38 (-0.47 to 1.24) | 1.48 (0.62 to 3.52) |
| 30-day major GUSTO bleeding^b^ | 7 | 0.2 | 9 | 0.2 | 0.04 (-0.27 to 0.35) | 1.15 (0.41 to 3.24) | 0.06 (-0.48 to 0.60) | 1.24 (0.20 to 7.80) |
| Optimal in-hospital medication^c^ | 1056 | 29.7 | 2432 | 54.7 | 19.57 (16.57 to 22.58) | 2.59 (2.30 to 2.91) | 23.56 (19.29 to 27.83) | 3.18 (2.63 to 3.84) |
| Optimal discharge medication^d^ | 1586 | 54.7 | 3182 | 78.1 | 21.75 (18.04 to 25.47) | 3.19 (2.79 to 3.63) | 23.55 (18.59 to 28.51) | 3.49 (2.82 to 4.32) |
| Tobacco cessation advice^e^ | 1367 | 96.1 | 975 | 93.3 | -1.14 (-3.38 to 1.10) | 0.82 (0.56 to 1.20) | 3.24 (-3.30 to 9.78) | 1.70 (0.63 to 4.55) |

Abbreviations: MACE: major adverse cardiovascular events defined as death, reinfarction, stroke, and major GUSTO bleeding.

^a^Odds ratios represent effect of intervention compared with control and are calculated as the difference in marginal effects (intervention group minus control group) in a mixed-effects logistic regression model including a random-effects term to account for within-hospital clustering. Primary analysis additional accounted for temporal trends.

^b^Major bleeding is defined by the Global Utilization of Streptokinase and Tissue Plasminogen Activator for Occluded Coronary Arteries (GUSTO) criteria, which is defined by intracerebral haemorrhage or bleeding resulting in substantial hemodynamic compromise requiring treatment.

^c^Composed of aspirin, adenosine diphosphate receptor antagonist (clopidogrel, prasugrel, or ticagrelor), anticoagulant, and β-blocker among patients eligible to receive all medications.

^d^Composed of aspirin, adenosine diphosphate receptor antagonist (clopidogrel, prasugrel, or ticagrelor), statin, and β-blocker among discharged patients eligible to receive all medications.

^e^Among discharged patients who reported smoking at baseline.

**Table S4.** Unadjusted and adjusted primary and secondary trial outcomes using mixed effect logistic regression models that account for within-hospital clustering and clustering and temporal trends among sites that implemented **2 or fewer ACS QUIK toolkit intervention components.**

| **Outcome** | **Control**  **(N=1359)** | | **Intervention**  **(N=2991)** | | **Cluster Adjusted Difference,**  **% (95% CI)**^a^ | **Cluster Adjusted**  **OR (95% CI)**^a^ | **Primary Analysis Difference,**  **% (95% CI)**^a^ | **Primary Analysis**  **OR (95% CI)**^a^ |
| --- | --- | --- | --- | --- | --- | --- | --- | --- |
|  | **n** | **%** | **n** | **%** |  |  |  |  |
| *Primary Outcome* |  |  |  |  |  |  |  |  |
| 30-day MACE | 132 | 9.7 | 190 | 6.4 | -1.93 (-3.56 to -0.30) | 0.73 (0.57 to 0.93) | -2.03 (-5.30 to 1.24) | 0.72 (0.44 to 1.17) |
| *Secondary Outcomes* |  |  |  |  |  |  |  |  |
| 30-day mortality | 95 | 7.0 | 145 | 4.8 | -1.22 (-2.62 to 0.18) | 0.77 (0.58 to 1.02) | -0.86 (-3.51 to 1.78) | 0.83 (0.48 to 1.43) |
| 30-day cardiovascular mortality | 94 | 6.9 | 141 | 4.7 | -1.21 (-2.57 to 0.15) | 0.76 (0.58 to 1.01) | -0.51 (-3.00 to 1.98) | 0.89 (0.51 to 1.55) |
| In-hospital mortality | 67 | 4.9 | 106 | 3.5 | -0.74 (-1.88 to 0.41) | 0.80 (0.58 to 1.11) | -2.89 (-6.12 to 0.33) | 0.48 (0.25 to 0.91) |
| 30-day re-infarction | 34 | 2.5 | 41 | 1.4 | -0.38 (-1.22 to 0.46) | 0.77 (0.48 to 1.24) | -0.06 (-1.90 to 1.79) | 0.96 (0.26 to 3.60) |
| 30-day stroke | 6 | 0.4 | 23 | 0.8 | Non-estimable | Non-estimable | Non-estimable | Non-estimable |
| 30-day major GUSTO bleeding^b^ | 1 | 0.1 | 12 | 0.4 | Non-estimable | Non-estimable | Non-estimable | Non-estimable |
| Optimal in-hospital medication^c^ | 349 | 26.6 | 468 | 16.3 | -20.11 (-25.57 to 14.65) | 0.31 (0.25 to 0.38) | -20.07 (-27.24 to -12.90) | 0.31 (0.22 to 0.44) |
| Optimal discharge medication^d^ | 729 | 57.8 | 1360 | 49.4 | -3.84 (-6.66 to -1.01) | 0.81 (0.70 to 0.94) | -4.22 (-9.26 to 0.82) | 0.79 (0.60 to 1.05) |
| Tobacco cessation advice^e^ | 505 | 95.3 | 731 | 96.4 | 4.99 (-0.43 to 10.40) | 2.12 (1.08 to 4.14) | -6.74 (-13.73 to 0.25) | 0.29 (0.08 to 0.98) |

Abbreviations: MACE: major adverse cardiovascular events defined as death, reinfarction, stroke, and major GUSTO bleeding.

^a^Odds ratios represent effect of intervention compared with control and are calculated as the difference in marginal effects (intervention group minus control group) in a mixed-effects logistic regression model including a random-effects term to account for within-hospital clustering. Primary analysis additional accounted for temporal trends.

^b^Major bleeding is defined by the Global Utilization of Streptokinase and Tissue Plasminogen Activator for Occluded Coronary Arteries (GUSTO) criteria, which is defined by intracerebral haemorrhage or bleeding resulting in substantial hemodynamic compromise requiring treatment.

^c^Composed of aspirin, adenosine diphosphate receptor antagonist (clopidogrel, prasugrel, or ticagrelor), anticoagulant, and β-blocker among patients eligible to receive all medications.

^d^Composed of aspirin, adenosine diphosphate receptor antagonist (clopidogrel, prasugrel, or ticagrelor), statin, and β-blocker among discharged patients eligible to receive all medications.

^e^Among discharged patients who reported smoking at baseline.
