# Supplementary material for: Implementation and acceptability of a heart attack quality improvement intervention in India: a mixed methods analysis of the ACS QUIK trial
Source: Implement Sci. 2019 Feb 6;14:12. doi: 10.1186/s13012-019-0857-7 (PMC6364470; doi:10.1186/s13012-019-0857-7)

Additional file 4: Figure S2**.** Rate of major adverse cardiovascular events at 30 days in the intervention and control groups within the ACS QUIK trial, as well as adjusted difference and adjusted odds ratio (95% confidence intervals) stratified by sites by participation in process evaluation interviews and number of quality improvement toolkit intervention components implemented.


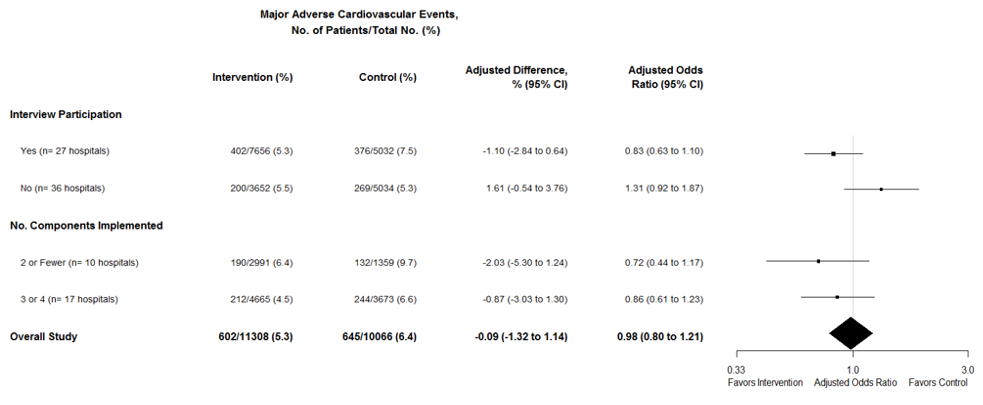

Supplement: Supplementary file 4 — Figure S2. Rate of major adverse cardiovascular events at 30 days in the intervention and control groups within the ACS QUIK trial, as well as adjusted difference and adjusted odds ratio (95% confidence intervals) stratified by sites by participation in process evaluation interviews and number of quality improvement toolkit intervention components implemented (DOCX 111 kb) [file 13012_2019_857_MOESM4_ESM.docx]
